# Tracheal Regeneration: Recent Progress in the Application of Stem Cells in Tracheal Bioengineering

**DOI:** 10.3390/ijms27062891

**Published:** 2026-03-23

**Authors:** Fatemeh Ganji, Florian Le Billan, Siba Haykal, Golnaz Karoubi

**Affiliations:** 1 Latner Thoracic Research Laboratories, University Health Network, Toronto, ON M5G 1L7, Canada; 2 Division of Plastic and Reconstructive Surgery, Department of Surgery, Yale New Haven Hospital, Yale School of Medicine, New Haven, CT 06510, USA; 3 Department of Mechanical and Industrial Engineering, University of Toronto, Toronto, ON M5S 1A1, Canada; 4 Department of Laboratory Medicine and Pathobiology, University of Toronto, Toronto, ON M5S 1A1, Canada

**Keywords:** trachea regeneration, bioengineered grafts, pluripotent stem cells, recellularization

## Abstract

Traumatic injury, stenosis, and malignancy involving large segments of the airway are difficult to reconstruct and require novel solutions. Despite advances in surgical techniques, the reconstruction of long-segment tracheal defects remains a significant challenge. Several bioengineering approaches have been explored for tracheal regeneration in vitro and in vivo, using cells in combination with three dimentional (3D) biological or synthetic scaffolds. This paper reviews recent advances in developing bioengineered trachea and the technologies utilized toward generating transplantable tracheal grafts. Specifically, the review will focus on the recellularization of tissue-engineered grafts using natural or synthetic scaffolds, highlighting relevant cell types used to reconstitute tracheal epithelium and cartilage. The promise of newly explored paradigms, including the application of pluripotent stem cells, will be discussed with an overview of associated challenges and necessary steps for future translation. Overall, these advances provide a foundation for the development of clinically viable tracheal grafts, bringing engineered tracheal reconstruction closer to reality.

## 1. Introduction

The trachea is a semi-flexible tube composed of 20 incomplete C-shaped outer rings of hyaline cartilage, middle connective tissue, and an inner epithelial layer [1]. The cartilage tissue provides mechanical strength, preventing tracheal collapse during breathing. The internal surface of the trachea is lined with pseudostratified mucociliary epithelium composed of multiciliated, goblet and basal cells, which serve as a barrier and help remove particles and debris from the airway [2,3]. Tracheal dysfunction is associated with damage to the epithelium and cartilage, resulting from surgical injury, tumor, infection, and prolonged intratracheal intubation. Extensive damage leads to tracheal stenosis, tracheomalacia, and airway collapse [4,5]. Despite advances in surgical techniques in recent decades, long-segment tracheal defects remain a significant challenge. To address this challenge, many research groups are exploring bioengineering approaches as an alternative technology for tracheal regeneration in vitro and in vivo, using autologous cells in combination with 3D biological or synthetic scaffolds [6]. While there is a considerable amount of literature focusing on the use of synthetic materials for tracheal graft generation [7,8], the choice of material alone is insufficient; successful grafts require functional cells to restore airway structure and function. This review will focus predominantly on the use of natural biomaterials in combination with various cell sources.

Bioengineered tracheal grafts require two main components: cartilage and the epithelium. Over the last several years, there has been significant effort put forth toward achieving a transplantable bioengineered tracheal graft. While progress has been made, research in the tracheal tissue engineering field has shown that there are considerable challenges that impede adequate cartilage and epithelial regeneration [9,10,11,12]. Perhaps one of the most critical limitations in tracheal tissue engineering is the lack of a functional epithelium. Indeed, preclinical and clinical studies demonstrated that the absence of epithelium leads to airway stenosis and obstruction, resulting in graft failure [13,14,15]. Consequently, recent research in the field has focused on the recellularization of the tissue-engineered grafts that has been shown to accelerate tissue regeneration [16,17,18,19].

Various cell types have been employed for the recellularization of tracheal grafts. These include cell lines [19,20], primary epithelial cell populations such as tracheal epithelial cells [21,22], nasal epithelial cells [11,23] and, more recently, pluripotent-derived cells [24,25]. Of particular interest are induced pluripotent stem cells (iPSCs), which have the capacity for self-renewal and differentiation—similar to that of embryonic stem cells (ESCs). These properties make them highly relevant for therapeutic applications [26,27]. IPSCs are advantageous in that they can be derived from various cell sources [28,29,30,31], lack the ethical complexities associated with ESCs, and can be patient-specific, thus evading immune rejection [32,33,34,35]. Recent advances in directed differentiation protocols toward generating airway epithelial cells have shown that iPSCs can differentiate into a mature airway epithelium composed of ciliated, goblet and basal cells [36,37,38,39,40,41], making them ideal for the recellularization of tracheal grafts. Here, we review advances in tracheal tissue engineering and focus on recent progress of the application of iPSCs in the generation of tracheal grafts.

## 2. Clinical Need and Current Approaches for Tracheal Repair and Replacement

Tracheal dysfunction or long-segment tracheal defects can be either congenital or secondary due to trauma, infection, or several types of cancer. An estimated 2.5 to 3.2% of trauma-associated morbidity and mortality results from tracheal injury [5]. In adults, malignant tumors in the airways cause tracheal stenosis and obstruction that require tracheal resection and reconstruction [42,43]. In intensive care unit patients or COVID-19 patients, it has been shown that prolonged endotracheal intubations cause laryngotracheal injuries and stenosis after extubation [44,45,46]. Currently, surgical techniques such as end-to-end anastomosis and surgical reconstruction of the trachea are the gold standard for the treatment of tracheal injuries in clinic but are not adequate for damage to the trachea that exceeds 50% of the tracheal length in adults and 30% of the tracheal length in children [5,47]. For long-segment defects, increased anastomotic tension and serious complications can exuberate injury [48,49]. Additionally, in circumstances in which the defect includes both the larynx and the trachea or is very close to the glottal area, end-to-end anastomosis is not ideal [50]. In these cases, the only viable treatment option is circumferential tracheal transplantation in which longer segments are reconstructed [51,52,53].

Currently, circumferential tracheal transplantation involved a two-stage surgical technique: the heterotopic/orthotopic allotransplantation. This involves placement of the donor trachea first in a highly vascularized heterotopic region of the recipient (such as the forearm) for an extensive period (weeks to months), which is followed by its subsequent transfer into an orthotopic position [54]. While tracheal allograft transplantation has been applied in the clinic and led to significant advances in treating long-segment tracheal defects [55,56], limitations remain that prevent graft functionality. These include the possible formation of fibrosis and stenosis, the induction of immune response, a lack of neovascularization, and inadequate epithelial regeneration and function in transplanted grafts [52,57,58]. Moreover, allograft transplantation requires an availability of donor tissues, is often associated with multiple surgeries, and it is also is very invasive. This significantly strains patient quality of life. Therefore, the use of naturally derived biomaterials to develop a biocompatible transplantable graft with low immunogenicity is an exciting alternative that may address some of these limitations [59,60,61,62].

## 3. Natural Biomaterials and Decellularized Tracheal Tissue Scaffolds for Bioengineered Grafts

Natural biomaterial-derived hydrogels are classified as protein-based (e.g., gelatin, collagen) and polysaccharide-based (e.g., agarose, alginate, starch) hydrogels [63]. Commonly used natural biomaterials in trachea regeneration include collagen, chitosan, gelatin, fibrin glue and silk fibroin. They allow the generation of porous scaffolds loaded with cells, and allowed cell growth and proliferation [64]. Table 1 summarizes reports using naturally derived biomaterials for use in tracheal regeneration. In parallel to the use of simple natural biomaterials, there have been significant efforts placed on using decellularized biological tracheal scaffolds.

**Figure 1:**
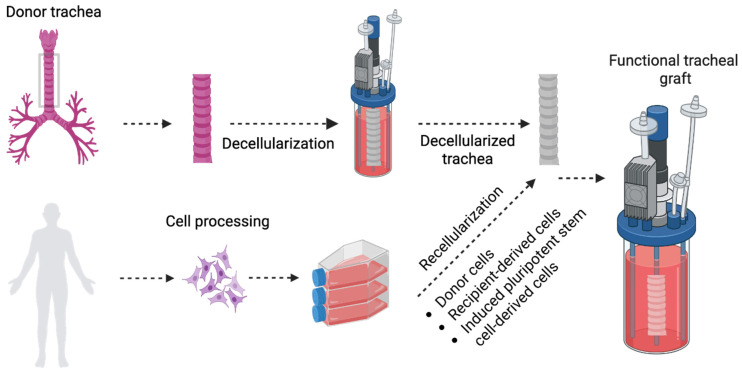
Bioengineering approaches in tracheal tissue engineering. Schematic illustration showing a general overview of tracheal tissue engineering highlighting the use of decellularized tissues. Decellularized tracheal grafts have been recellularized with various cell types including autologous cells derived from the patient’s own tissues, allogeneic cells from donors, and iPSC-derived cells to construct functional tracheal grafts.

Over the past decade, the decellularization of animal tracheal grafts, to generate naturally occurring acellular biological scaffolds, has received considerable attention (Figure 1). Using this approach, a biological scaffold is obtained by the full [65] or partial [11,66,67] removal of cells and cellular debris from the tissue while preserving the three-dimensional extracellular matrix (ECM) and its components. The main advantage of acellular biological scaffolds is the preservation of the microstructure of native tissue provided by the ECM along with signaling molecules, as it facilitates cell attachment, growth and differentiation [65,68]. Numerous decellularization protocols have been developed by combining physical, chemical, and/or enzymatic approaches [69]. Their common objective is to achieve the partial or complete removal of native cells while minimizing harmful effects on the ECM structure and composition [70,71,72]. And complete removal of the cells has been shown to result in damage to the tracheal tissue and a decline of mechanical strength [72,73]. To circumvent these challenges, many investigators pivoted research toward the use of recellularized grafts via repopulation of the lumen, cartilaginous layer or both [11,20,59].

**Table 1:** Natural biomaterials and biological scaffolds for bioengineered tracheal grafts.

Scaffold/ Material	Graft Size	Cell Source	Experimental Details	Outcomes	Study
**Acellular tracheal patch allografts**	Patch 15 × 15 mm	No cells	Pigs (4w) were transplanted with acellular allogeneic tracheal grafts followed by bronchoscopic and histological evaluation at 11 weeks post-transplantation.	Acellular grafts showed some re-vascularization and re-epithelialization after implantation with regeneration of cartilage foci adjacent to the grafts. However, grafts failed due to stenosis and collapse.	[74,75]
**Acellular and recell-** **ularized tracheal allografts**	Long-segment circumferential graft 2–3 cm	Autologous mesenchymal stromal cells (MSCs) and tracheal epithelial cells	Porcine acellular and recellularized long-segment circumferential tracheal allografts heterotopically transplanted and followed by evaluation of in vitro and in vivo T cell proliferation and infiltration.	Acellular graft delayed leukocyte infiltration, but eventually cartilage degradation was observed due to the incomplete removal of MHC in the submucosal gland and CD4^+^ T cells induction. Recellularization induced immunological tolerance by the recruitment of CD4^+^CD25^+^Foxp3^+^ regulatory T cells.	[59]
**Partially decellularized acellular** **trachea**	Long-segment graft 3–4 mm	No cells	Heterotopic transplant of mice acellular trachea was evaluated at 28 days post-implantation. Restoration of the tracheal micro- vasculature was quantified by counting CD31^+^ cells within the submucosa. Graft patency was assessed in vivo with micro-computed tomography.	Implantation of partial acellular grafts resulted in the restoration of native tracheal rigidity, high chondrocyte viability, neo-epithelialization and endothelialization at endpoint.	[60]
**Acellular tracheal** **allografts**	Long-segment graft 5 × 5 mm	Canine tracheal epithelial cells and canine yolk sac endothelial progenitor cells (YS)	Recellularized canine trachea scaffolds fragments were implanted subcutaneously into the right and left medial dorsal thoracic region of nude mice. Control group received acellular scaffold.	Implanted tissue remained preserved and proliferative, and no fibrotic tissue process was observed in animals after 45 days. Differentiation ability was shown through the CK18 and β-tubulin expression in epithelial cells.	[22]
**Combination of** **acellular tracheal** **and acellular hum-** **an dermis**	Long-segment graft 5 cm	Human bronchial epithelial cells (HBECs) and lung fibroblasts	Tissue-engineered respiratory mucosa was generated via recellularization of human dermis (HBECs and lung fibroblasts) in air liquid interface (ALI) culture system and subsequently engrafted on pre-vascula-rized, acellularized trachea and hetero- topically implanted into immunosuppressed rabbits and nude mice.	Integration of graft with sign of re-vascularization was observed as well as infiltration of inflammatory cell. In vivo experiment demonstrated survival and retention of epithelial cells that expressed pan-keratin.	[61]
**Acellular tracheal graft**	Fully circumferential graft 5–7 rings length	Mouse iPSC-derived definitive endoderm cells	Recellularized rat tracheal segments (7 rings) were orthotopically transplanted on nude rats.	Luminal surface of graft re-epithelialized with numerous ciliated epithelial cells. Animals died after 5 weeks due to airway stenosis.	[20]
**Partially decellularized autograft**	Patch 1 × 1 cm	Autologous nasal epithelial cell sheet	Cell sheets were prepared via seeding porous polyethylene terephthalate (PET) membranes. Defects were made via removal of epithelium in situ. Sheets were applied onto the luminal surface of the decellularized graft immediately re-transplanted into the original defect. Control group was transplanted with decellula-rized trachea without cell sheet application.	At two months post-operation, orthotopic transplantation of graft indicated integration of graft into the host trachea with healing of luminal surface. In control groups, epithelial hypertrophy, fibroproliferation and neovascularization in sub-epithelial layer was observed.	[11]
**Partially decellularized tracheal scaff-** **old (porcine trachea)**	Circumferential tracheal graft (~5 cm)	No cells	Partially decellularized tracheal segments were implanted in a porcine model and evaluated by bronchoscopy, imaging, and histology at day 28 and day 56 post- implantation to assess biointegration.	The scaffold showed progressive biointegration with neovascularization, fibroblast colonization of the scaffold, no infection or tissue necrosis and no significant graft rejection at day 28. However, cartilage regeneration remained limited and structural integrity was not fully restored at day 56	[76]
**Polypropylene mesh and collagen sponge**	Patch 12 mm in diameter	Mouse iPSCs-derived epithelial cells	5-day-old iPSCs-derived embryoid bodies (EBs) were cultured at ALI model for 28 days. EBs containing ciliated-like structures were loaded on collagen-coated artificial tracheal graft followed by orthotopic transplantation into nude rats.	Effective coverage of artificial material with differentiated epithelium with γ-tubulin expression on the basal sides of ciliated cells after 7 days. Only 50% of nude rats used for the experiment showed iPSCs-derived tissues.	[77]
**Collagen, polypropylene mesh and** **collagen vitrigel membrane**	Patch 12 mm in diameter	Human iPSC-derived multiciliated airway cells (MCACs)	42-day-old proximal airway progenitor cells were cultured on collagen vitrigel membrane under ALI condition to create cell sheet. Internal side of an artificial trachea consisting of polypropylene mesh and collagen sponge was covered by hiPSC-MCACs sheet and transplanted into a nude rat trachea.	The survival of transplanted cells in the endotracheal epithelium of tracheal defect was observed at 1 and 2 weeks after transplantation and hiPSC—MCACs displayed motile cilia on collagen vitrigel membrane.	[24]
**Silk fibroin and collagen vitrigel membrane (SF-CVM)**	Patch 2.5 cm in diameter	Human iPSC-derived airway epithelial cells	Fabricated SF-CVM was glued onto trans-well inserts using fibrin glue, rehydrated and seeded with airway progenitors. Subsequent culture in ALI achieved functional mucociliary epithelia. hiPSC-derived SF-CVM grafts were evaluated in ex vivo and in porcine tracheal defects.	Differentiation of mucociliary epithelium was achieved on SF-CVM graft after 3 days. Integration with host tracheal tissue was observed as well as no airway collapse. SF-CVM graft was covered with hiPSC-derived p.seudostratified epithelium (~80%) on day 3 with reduction in granulation tissue formation.	[25]
**Core–shell poly (L-lactic acid-co-Ɛ-caprolactone)/collagen scaffolds containing TGF-β3**	Patch 1 × 2 cm	Rabbit mesenchymal stromal cells (MSCs)	Electrospun PLCL–collagen membrane with a ratio of 75:25 containing TGF-β3 was spread on 6 3D-printed grooved PLCL molds (6 mm diameter) each seed-ed with cells. Molds were stacked and cultured for 7 days and then implanted into rabbit tracheal defects.	Sustained release of TGF-b3 scaffolds induced the differentiation of chondrogenic cells. Tracheal integrity was maintained for 2 months after restoration; meanwhile, the entire luminal surface of the engineered patch was re-epithelialized.	[78]
**Porcine atelocoll-** **agen (I and III) sponge and poly** **propylene framework**	Patch tracheal defect size (2 mm wide, over two cartilages)	Autologous adipose tissue-derived stem cells (ASCs)	Collagen sponge containing polypropylene mesh was prepared and used as tracheal graft. The collagen solution containing ASCs was stratified on the tracheal graft and then incubated for 1 h to form a collagen gel. Stratified trachea graft was transplanted into porcine tracheal defect.	ASC transplantation after 3 weeks promoted ciliogenesis with ciliary function comparable to the normal rat trachea.	[62]
**Polycaprolactone (PCL) and hydrogel**	Semi-circumferential 1.5 × 1.5 cm	Autologous nasal epithelial cells and chondrocyte cells	Artificial trachea (5 layers consisting of PCL, alginate with epithelial cells, PCL, alginate with chondrocytes and PCL). Grafts transplanted into rabbit tracheal defect for graft without cells used as a control.	Observation at 3, 6 and 12 months showed that artificial tracheas were effective in the regeneration of respiratory epithelium but not in cartilage regeneration.	[79]
**3D-printed polycaprolactone (PCL) backbone integrated with freeze-dried** **collagen–hyaluronic acid (CHyA) layer**	Tubular scaffold Inner diameter: 9.6 mm Outer diameter: 12 mm	Calu-3 respiratory epithelial cells and Wi38 lung fibroblasts	Scaffolds were fabricated using a 3D-printed PCL framework reinforced with a CHyA layer. Hybrid scaffolds facilitated spatially targeted seeding of epithelial cells on the luminal surface and fibroblasts on the outer layer.	PCL reinforcement significantly improved scaffold mechanical stability. Targeted seeding enabled spatial cell organization, resulting in successful epithelial coverage and sustained fibroblast viability, demonstrating a promising platform for tracheal tissue engineering.	[80]

## 4. Recellularization of Acellular Tracheal Grafts

The objective of recellularization is to recreate the microanatomy of the tissue and/or organ with the ultimate goal of creating a functional bioengineered graft. Recellularization requires the use of appropriate cell sources, effective seeding methods, and physiologically relevant cell culture methods usually carried out in customized bioreactor systems. In the case of the trachea, several bioreactor systems allow decellularization and/or recellularization [18,19,81,82]. Studies, including ours [59], have found that recellularization results in improved graft outcomes following transplantation [15,18,22]. In addition, ex vivo experiments have demonstrated that the resulting epithelium provides cues for the promotion of vascularization and angiogenesis [83,84].

There have been several cell sources used for the recellularization of tracheal grafts with the objective to reconstitute the epithelium and the cartilage (Table 2). Fully differentiated somatic cell types include chondrocytes [85,86,87,88,89,90] and epithelial cells [21,91,92,93]. Progenitor cell populations, that display greater plasticity, include subtypes of adult mesenchymal stem cells (bone marrow, adipose, umbilical cord blood and amniotic fluid-derived mesenchymal stem cells (MSCs)) [59,78,94,95,96,97,98]. More recently, iPSCs have been investigated as a cell source. These include iPSC-derived mesenchymal stem cells (iPSC-MSCs), iPSC-MSC-derived chondrocytes [99,100,101,102] and iPSC-derived epithelial cells [77,103]. These cells have the added advantage of allowing patient specificity for personalized bioengineered grafts [32,39,104,105,106,107,108]. Table 2 summarizes the different cell sources used for tracheal graft recellularization; we will discuss cell sources with a specific focus on the use of pluripotent stem cells.

**Table 2:** Cell sources for tracheal tissue engineering.

Cell Type	Tissue Source	Advantages	Limitations	Study
**Chondrocytes**	Auricular	Easily accessible	Limited purity and yield Limited expansion in culture Limited survival in vivo Difficult to harvest with limited yield and lower proliferation capacity	[86,109,110]
	Nasal septum cartilage	Phenotypically and functionally similar to tracheal chondrocytes		[88,111,112,113]
	Costal cartilage			[88]
	Tracheal cartilage	Ideal biomimicry		[87,112]
**Mesenchymal stromal cells**	Bone marrow-derived MSCs (BMSCs)	Good capacity to differentiate into chondrogenic lineages	Decreased proliferative and multipotent capacity with increasing donor age Can be differentiated to hyaline cartilage-like tissue derived from MSC. Limited capacity to keep phenotype and function Tendency of MSC to differentiate toward hypertrophic cartilage instead of hyaline cartilage MSC-derived chondrocytes upregulate type II collagen, proteoglycans and upregulation hypertrophy markers, such as type X collagen, and alkaline phosphatase	[114]
	Adipose-derived MSCs (AMSCs)	Easier to harvest and expand in vitro compared with BMSCs, and have multi-differentiation potential that is independent of age		[115]
	Umbilical cord blood-derived MSCs (UCB-MSCs)	Good capacity to expand and differentiate		[96,116]
	Amniotic fluid-derived MSCs (AFMSCs)	AFMSCs are able to differentiate into lineages of three germ layers and have no tumorigenicity in vivo unlike ES cells		[117,118]
**Epithelial cells**	Tracheal epithelial cells	Ideal biomimicry	Difficult to harvest in large numbers Limited proliferation and differentiation	[18,21,91]
	SMG-associated myoepithelial cells	Capacity to differentiate and reconstitute tracheal epithelium		
	Nasal turbinate epithelial cells	Similarities to tracheal epithelium Can be expanded in culture		
	Skin epithelial cells	Have capacity to transdifferentiate into tracheal epithelial cells and chondrocytes Skin epithelial cells are easily accessible and can be expanded to large numbers		[119]
**Epithelial stem cells**	Endogenous stem/progenitor cells present in the respiratory tract, such as ductal cells, basal cells and variant club cells	Able to differentiate into different types of tracheal epithelial cells No risk of tumor formation	Challenging to isolate and culture	[120]
**ESCs**	Pluripotent ES cells derived from the inner cell mass of the blastocyst	ES cells could generate a fully differentiated airway epithelium composed of basal, ciliated, intermediate and club cells	Ethical issues with ESCs given their source Immune rejection resulting from ESCs Differentiated cells derived from ESCs and iPSCs are often heterogeneous and purity is variable Risks of tumor formation in vivo	[121,122,123]
**iPSCs**	Derived from somatic cells which can be patient specific	Capable of differentiation into a variety of cell lineages Non-immunogenic cells No ethical issues		[20,24,25,77,100,101,124,125]

## 5. Recellularization Approaches for Cartilage Regeneration

Tracheal cartilage is a highly organized tissue in which complex structural extracellular networks enclose chondroblasts and chondrocytes [126]. Cells sparsely populate the tissue, which is otherwise composed of highly cross-linked extracellular matrix (10% aggrecan, 75% water, and a mix of collagen fiber and other constituents).

Primary chondrocytes are limited in their proliferative capacity and are prone to rapid changes in their phenotype in culture [127,128]. MSCs are the most commonly used cell type for recellularization of the tracheal cartilage, as they are easily accessible and expandable, and they display low immunogenic properties [129,130]. In addition, they produce a wide range of trophic and immunomodulatory factors [129,131], which enhance their therapeutic potential [101,132,133]. However, there are several disadvantages associated with the recellularization of grafts with MSCs. These include significant variability in their characteristics, and function in long-term culture [133,134,135]. In addition, grafts recellularized with MSCs do not fully recapitulate the mechanical properties of the native trachea [16,136].

## 6. Use of PSCs for Cartilage Regeneration

iPSCs have recently been explored as a source from which chondrocytes can be derived [137,138,139,140]. Pluripotent stem cells can be differentiated into chondrocytes as a monolayer when cultured in the presence of specific growth factors. For chondrogenesis via monolayer culture, after 1 week of culture in a pro-mesodermal medium (supplemented in BMP-4, PDGF-BB and FGF2), culture media is replaced with basic culture media supplemented in TGF-β3 for a subsequent week and with both TGF-β3 and IGF-1 for another week [141]. Other protocols involve the generation of embryoid bodies from ESCs or iPSCs, which give rise to mesenchymal stromal cell (MSC) outgrowths. MSCs are then selected via cell sorting and committed to a chondrocyte lineage using TGF-β3. Chondrocytes are evaluated by the expression of phenotypic markers type II collagen, aggrecan and Sox9 [142].

**Figure 2:**
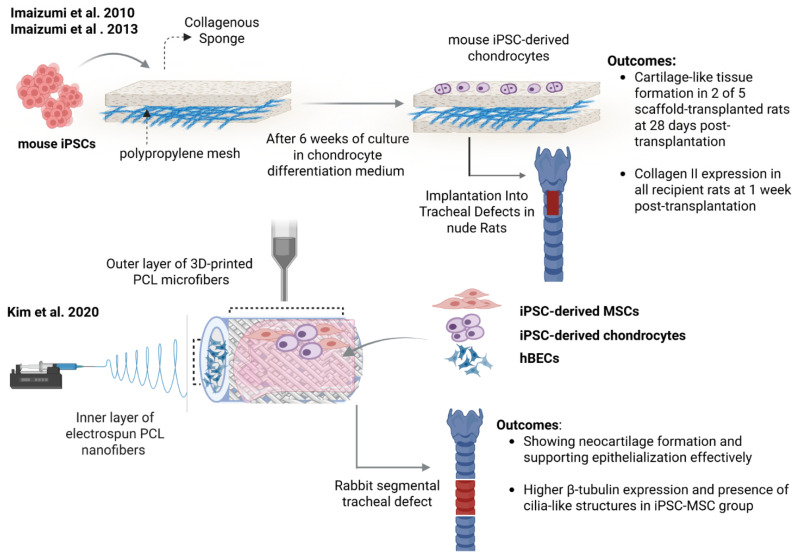
Strategies for repairing airway cartilage. (**Top**): Mouse iPSC-derived chondrocytes seeded on a polypropylene mesh with a collagenous sponge formed cartilage-like tissue after implantation in nude rats [100,124]. (**Bottom**): A bi-layered PCL scaffold seeded with iPSC-derived mesenchymal stem cells (MSCs), chondrocytes, and human bronchial epithelial cells (hBECs) promoted neocartilage formation and epithelialization in a rabbit model [101]. Created with BioRender.com (accessed on 5 February 2026).

In 2010, Imaizumi et al. reported the first application of iPSC-derived chondrocytes for the repair of a tracheal cartilage defect (Figure 2) [100]. Mouse iPSCs were seeded on collagen sponge/polypropylene mesh and differentiated for 6 weeks into chondrocytes. The mesh was then transplanted into tracheal defects (1.5 mm wide, 2.5 mm long) in a nude rat model. In vivo analysis at four weeks post-transplantation showed that a tenth of the original defect displayed the formation of cartilage-like tissue in two out of four rats. However, the regenerated tissue was relatively far from the defect area. Importantly, all transplanted rats revealed teratoma formation due to the presence of undifferentiated iPS cells. Limited regeneration was thought to be due to the poor differentiation of iPSCs into chondrocytes. A subsequent study from this group further characterized the differentiation of iPSC into chondrocytes at different time points both in vivo and ex vivo after transplantation in rats (Figure 2) [124]. Ex vivo assessment showed the expression of chondrocyte markers type II collagen and aggrecan at four weeks and S-100 protein at six weeks. The effects of the duration of iPSC differentiation into chondrocytes on cartilage formation were evaluated ex vivo one week or four weeks after transplantation. Results showed no cartilage formation in the animals transplanted with the three-week differentiated iPSCs. While in those transplanted with the six-week differentiated iPSCs, the positive expression of type II collagen, indicating cartilage regeneration, was observed in all recipients at one week after transplantation. However, at four weeks post-transplantation, only three out of eight rats showed cartilage formation within the edge of the scaffolds. The authors suggested that differentiated iPSCs might dedifferentiate or die in vivo over time. Despite areas of cartilage regeneration, results also showed teratoma formation in fourteen out of eighteen rats. The most recent study from the Omori group investigated the use of hiPSCs-derived MSCs, grown on their signature patch made of collagen sponge and polypropylene mesh, to regenerate cartilage. Twelve weeks after their transplantation into tracheal defects, three out of seven nude rats exhibited cartilage-like tissue generation [143].

Kim et al. reported the use of iPSC-derived MSCs and chondrocytes for the fabrication and transplantation of a double-layer tubular scaffold in a rabbit tracheal defect model (Figure 2) [101]. The tubular construct was composed of an inner layer of electrospun PCL nanofibers and an outer layer of 3D-printed PCL microfibers. Both layers were coated with matrigel to enhance cell attachment. Human bronchial epithelial cells (hBECs) were used to cover the inner layer, and human iPSC-derived mesenchymal stem cells (hiPSC-MSCs) or human iPSC-MSC-derived chondrocytes (iPSC-Chds) were seeded onto the outer layer. Constructs were transplanted in a rabbit segmental tracheal defect (1.5 cm long) and evaluated at four weeks post-transplantation. Hematoxylin and eosin (H&E) staining, safranin O staining results and micro-CT images revealed that groups receiving iPSC-Chds, and to a lesser extent iPSC-MSCs, were effective in cartilage formation compared to the acellular scaffold controls. Moreover, they found a significant decrease in mononuclear cells in the iPSC-MSC and iPSC-Chd groups compared with the control groups. The newly formed cartilage spread well along the 3D-printed mesh structure, especially in the iPSC-Chd group. Both experimental groups were also effective in supporting epithelialization with a thicker epithelial layer showing positive staining for keratin 5 and β-tubulin. β-tubulin expression was predominant in the iPSC-MSC group that also showed the presence of cilia-like structures. Importantly, the authors concluded that rather than replacement of the tracheal cartilage and regenerating the mucosal layer, hiPSC-MSCs, hiPSC-Chds, and hBECs are inducing remodeling through paracrine mechanisms involving cytokines, growth factors and ECM components.

Recent advancements in human ESC culture automation processes have allowed a larger-scale production of cartilage sheets. As an example, embryoid bodies derived from human ESCs were grown and differentiated on collagen with xeno-free chondrogenic media. After two months of automated culture, the resulting cell sheets were implanted subcutaneously in immunodeficient NOG mice. Another two months allowed the generation of mature fully differentiated cartilage sheets, and tests in preclinical models could prove promising [144].

Thus far, studies have shown that while promising, the use of iPSCs for cartilage regeneration is a highly complex and long process with several limitations. These include inefficiencies in the differentiation process leading to a variable number and size of embryoid bodies, composed of heterogeneous cell populations, and the presence of undesired cell lineages [145,146,147]. Given the existing challenges with cartilage regeneration, partial decellularization as an alternative has gained considerable attention. As such, tracheal grafts have been partially decellularized to remove only the epithelium, preserving chondrocytes [66]. Importantly, studies have shown that the cartilage, due to its dense matrix, is difficult to infiltrate and is thus immune-privileged and does not cause a significant immune response [148,149,150,151].

## 7. Recellularization Approaches for Epithelial Regeneration

**Figure 3:**
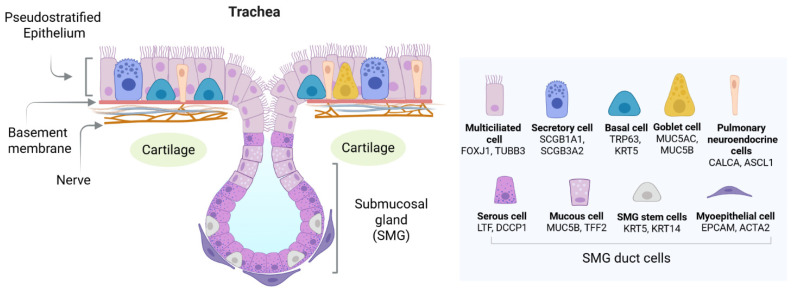
The cellular composition of tracheal epithelium. The cartilaginous airway is covered with a pseudostratified epithelium consisting of ciliated, secretory, basal and neuroendocrine cells. The proximal trachea is characterized by submucosal glands (SMGs) located between cartilage rings. The SMG ducts contain a population of stem cells that act as a stem cell reservoir for the tracheal epithelium. Created with BioRender.com (accessed on 5 February 2026).

The pseudostratified columnar epithelium of the proximal airways (trachea and main stem bronchi) is mainly composed of multiciliated, secretory cells with less represented populations of club cells, neuroendocrine cells (NECs), ionocytes, tuft cells, deuterosomal cells, and basal cells (BCs) (Figure 3) [152,153]. The proportions of goblet, secretory and basal cells vary along the proximal–distal axis. Airway goblet cells secrete mucus while ciliated cells, with their motile cilia lining the epithelial cells of the conductive airways, orchestrate mucociliary clearance [154]. Together with basal cells (the main progenitors of the airway epithelium), these cell populations are critical for the generation of a functional re-epithelialized tracheal graft.

Many groups have explored the potential of epithelial cells in accelerating tracheal regeneration by culturing these cells on the luminal surface of tracheal scaffolds ex vivo prior to transplantation [24,79,101]. The source of cells for recellularization is an important factor to consider. Primary tracheal epithelial cells are commonly used, as they have the ability to reconstitute the pseudostratified columnar epithelium, giving rise to the required goblet and ciliated cells (as in the native trachea) [21]. Additional primary epithelial cells have also been explored, such as those of the buccal mucosa and skin [18,119]. These studies have been summarized in Table 2. Despite promising proof-of-concept to date, primary epithelial cell sources are difficult to isolate and grow in culture, and they show high heterogeneity in differentiation capacity across donors with rapid changes in phenotype and function [155,156,157]. PSCs can self-renew unlimitedly and have the remarkable potential to differentiate into almost any cell type in the body. Therefore, they have been considered in developmental biology research, disease modeling, and regenerative medicine.

## 8. Use of PSCs for Epithelial Regeneration

**Figure 4:**
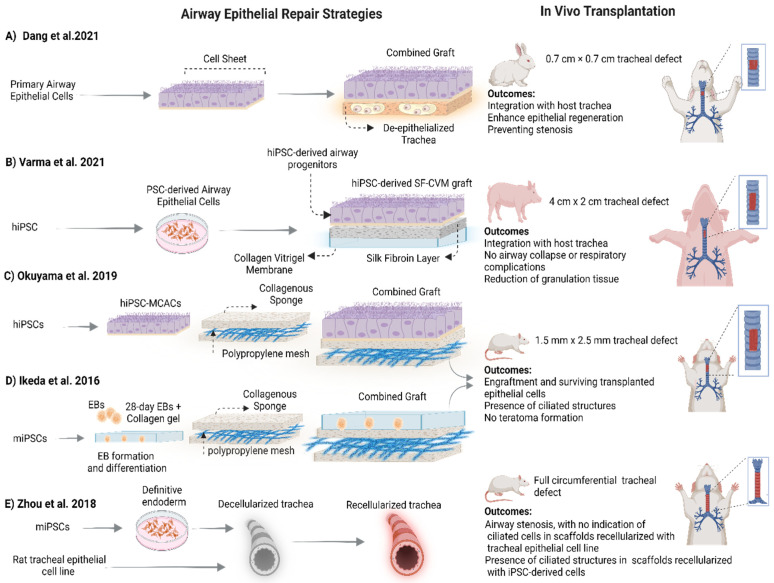
Strategies for repairing airway epithelium. This schematic summarizes recent strategies for tracheal epithelial regeneration using stem-cell-derived or primary airway epithelial cells combined with natural or synthetic scaffolds. (**A**) A sheet of epithelial cells generated from primary airway epithelial cells was combined with a de-epithelialized trachea and then transplanted into rabbits with a 0.7 × 0.7 cm tracheal defect [11]. (**B**) Human-induced pluripotent stem cell (hiPSC)-derived lung progenitors were combined with a composite biomaterial of silk fibroin and collagen vitrigel membrane (SF-CVM); then, they were followed with a preclinical application of graft in large animals with a 4 × 2 cm tracheal defect [25]. (**C**) An airway patch loaded with hiPSC-derived multiciliated airway cells (hiPSC–MCACs) was transplanted into nude rats with a 1.5 × 2.5 mm tracheal defect [24]. (**D**) Mouse iPSC-derived airway cells (day28 EBs) were loaded onto collagen I/polypropylene mesh scaffolds and transplanted into **a** nude rat tracheal defect model [125]. (**E**) A mouse iPSC-derived definitive endoderm and rat tracheal epithelial cell line were used to recellularize decellularized circumferential tracheal scaffolds and transplanted into a nude rat tracheal defect model [20]. Images were generated based on the information provided in the papers. Created with BioRender.com (accessed on 6 February 2026).

Over the past few years, there has been significant interest in the application of PSCs for the repair and regeneration of the tracheal epithelium [24,25,40,77]. Several research groups have demonstrated the differentiation of PSCs into airway epithelial cells [39,40,107,158,159,160,161]. Differentiation protocols have recently been reviewed (see Varma et al. [162]). In brief, proximal lung epithelial cells are derived from PSCs via stepwise mimicry of the developmental signaling pathways beginning with differentiation to definitive endoderm (SOX17^+^ and FOXA2^+^), anteriorization to foregut endoderm (SOX2^+^FOXA2^+^) and subsequent ventralization to create NKX2-1^+^ lung progenitors. Lung progenitors obtain a proximal fate via the upregulation of *SOX2*, *P63* and *SCGB2A2*, and they subsequently mature into multiciliated epithelium when exposed to air, which is typically via an air–liquid interface (ALI) culture system [162]. Here, we will summarize the current advances in generating tracheal grafts with the use of PSC-derived epithelial cells and their assessment in tracheal defect models (Figure 4).

The first use of PSCs in a tracheal scaffold was described by the Omori group [124]. The authors looked histologically and quantitatively at the propensity for teratoma formation after implantation into tracheal defects (1.5 mm × 2.5 mm) and abdominal subcutaneous tissue in rats. They employed mouse (iPS-MEF-Ng-20D-17) iPSCs in artificial tracheal scaffolds, consisting of collagenous sponge and polypropylene mesh. Specifically, undifferentiated mouse iPSCs suspended in collagen type I were introduced into artificial tracheas and cultured for six weeks under various media conditions (DMEM, DMEM + bone morphogenetic protein-2 (BMP-2), or chondrocyte differentiation medium). Histological evaluation showed the presence of undifferentiated cell colonies in all conditions after six weeks of ex vivo culture. To investigate site-dependent differences in teratoma formation, the cell-loaded artificial scaffolds were then transplanted into the cervical tissue around tracheal defects and in the abdominal subcutaneous tissue of nude rats. Four weeks after transplantation, teratoma formation was observed in both tracheal defect and subcutaneous areas with a higher number of teratomas in the cervical area in comparison to the abdominal subcutaneous tissue [103].

In later studies using the same model, this group reported that further differentiation of iPSCs via exposure to air-liquid interface (ALI) led to a significant reduction in the number of undifferentiated cells and reduced the risk of teratoma formation [77,125]. For these studies, iPSCs-derived embryoid bodies (EBs) were first cultured in differentiation medium for five days, which was followed by ALI culture for twenty-six days. In vitro examination over the ALI culture period showed the presence of conduit structures lined with epithelial cells as early as twelve days. At twenty-six days, ciliated-like structures appeared inside the conduits positive for β-tubulin IV (ciliated cell marker) and ZO-1 (tight junction formation marker). At the mRNA level, the authors observed increased expression of *β-tubulin IV* and *Forkhead box protein J1* (*FoxJ1*) genes as well as reduced expression of *Nanog* (marker of undifferentiated PSC). ALI-conditioned EBs were then loaded onto their previously described artificial tracheal scaffold (12 EBs per scaffold) and transplanted into a tracheal defect in nude rats for 1 week. In vivo examination showed that the luminal side of the tracheal defect was covered with stratified epithelium and indicated the presence of ciliated structures on grafts expressing β-tubulin IV and ZO-1 [125]. While promising, the authors showed that the integration of iPSCs-derived tracheal epithelium into an artificial scaffold was challenging, and only 50% of nude rats used for the experiment showed iPSCs-derived tissues [77].

The authors, in a subsequent study, used tdTomato-labeled mouse iPSCs to distinguish undifferentiated iPSCs and iPSC-derived epithelial cells and better delineate the integration of transplanted mouse iPSCs [163]. Labeled iPSCs were subjected to EB formation and ALI culture, showing that tdTomato-labeled iPSC-derived cells co-expressed *tdTomato* and *FoxJ1* after three weeks of expansion. Gene expression analysis confirmed the expression of tracheal epithelial markers including *mucin 5AC* (*Muc5ac*), *keratin 5* (*Krt5*) and *Tubulin beta-4a* (*Tubb4a*) on day twenty-one. Labeled iPSC-derived airway cells (day twenty-six EBs) were loaded onto their collagen I/polypropylene mesh scaffolds and transplanted in nude rats. Results at one week post-transplantation showed tdTomato- and Krt5-positive cells in a section of the tracheal defects. Furthermore, cells derived from labeled iPSCs were clearly distinct from recipient basal cells, suggesting the potential for iPS-derived cells in reconstituting the epithelium [20]. The above-summarized studies all used mouse-derived pluripotent cells. With progress in directed differentiation protocols for epithelium derived from human PSCs, recent studies have also shown the application of human iPSC-derived epithelial cells.

The Omori group also investigated the feasibility of transplanting an airway patch loaded with human iPSCs (hiPSC)-derived multiciliated airway cells (hiPSC–MCACs) in their nude rat tracheal defect model [24]. They used the same artificial graft consisting of polypropylene mesh and collagen sponge. First, day forty-two proximal airway progenitor cells were cultured on collagen vitrigel membranes under ALI conditions to create a cell sheet. The internal side of an artificial trachea was covered by a hiPSC–MCACs sheet together with a collagen vitrigel membrane and implanted in a nude rat model of tracheal defect. In vivo analysis confirmed that the tracheal defect was covered with a thick layer of epithelia and presented hiPSC-derived ciliated cells for up to two weeks after transplantation. Importantly, the survival and stability of the hiPSC-derived cell sheets were observed with high efficiency (all seven animals at two weeks after transplantation) [24]. In order to ameliorate the cellular engraftment, the Omori group further investigate the optimal duration of hiPSC differentiation [164]. They tested thirty-eight, forty-five, and fifty-six days of differentiation from hiPSCs to induced airways epithelial cells (iAECs). The survival rate of iAECs differentiated from hiPSC at fifty-six days was greater than that in the forty-five day group, which was itself higher than that in the thirty-six-day group. They further analyzed the proportions of differentiated cell types within the tracheal patch two weeks after transplantation within tracheal defects in nude rats. Proportions of hiPSCs-derived differentiated ciliated cells, basal cells, club cells, and goblet cells are close to the ones found in a native healthy human tracheal epithelium [165]. Recently, this group challenged the use of nude rats as a suitable immunodeficient preclinical model and led a comparative study including X-linked severe combined immunodeficiency (X-SCID) rats. In both rat strains, tracheal defects were surgically created and replaced by a patch of collagen sponges and polypropylene mesh seeded with hiPSCs-derived AECs. Two weeks post-transplantation, cell engraftment was confirmed in both rat strains. However, all subtypes of CD3-positive immune cells were in significantly lower amounts in the X-SCID rats compared to the nude ones [166]. With a decreased immune reaction, X-SCID could represent a new preclinical option for a trachea-humanized rat model.

In our own studies, we developed a composite biomaterial of silk fibroin and collagen vitrigel membrane (SF-CVM), supporting an enhanced maturation of epithelial cells into ciliated cells and displaying desirable mechanical characteristic [167]. We investigated the preclinical application of SF-CVM-based tracheal graft loaded with human-induced pluripotent stem cell-derived airway epithelial cells in large animal airway defects. hiPSC-derived airway progenitors achieved differentiation into the mucociliary epithelium on an SF-CVM graft with physiological proportions of ciliated and goblet cells. They also proved good integration with host tracheal tissue when implanted into porcine airway defects, while there was no airway collapse. In addition, the majority (~80%) of the SF-CVM graft was covered with hiPSC-derived pseudostratified epithelium three days post-operatively, which reduces the inflammatory responses and granulation tissue formation [25]. Overall, the outcomes revealed a significant differentiation of ciliated cells from iPSCs compared to primary human tracheal epithelial cells, and they demonstrated the clinical potential of biological patches loaded with iPSC-derived cells to induce the re-epithelialization in vivo as well as their ability to maintain airway patency.

To the best of our knowledge, there is currently only one study investigating PSCs for tracheal regeneration in a full circumferential transplantation model [20]. Specifically, Zhou et al. compared the potential of a rat tracheal epithelial cell line (EGV-4T) and two mouse iPS cell lines for the recellularization of the luminal surface of a rat decellularized circumferential tracheal scaffold (5-rings long). The orthotopic transplantation of scaffolds recellularized with EGV-4T cells (n = 2) showed no granulation tissue, and recipient rats survived for thirty days. However, wheezing occurred immediately after transplantation, and the animals displayed a decrease in body weight. In addition, airway stenosis was observed with cell infiltration in the submucosa with no indication of ciliated cells. In the iPSCs group, GFP-labeled iPS cells pre-differentiated into a definitive endoderm (Sox17^+^) were used for the recellularization of decellularized rat tracheas. Recellularized tracheal scaffolds were orthotopically transplanted into nude rats. All rats survived at least four weeks post-transplantation but showed wheezing and dyspnea. Four out of five rats suffered airways stenosis caused by an extensive proliferation of contaminating undifferentiated iPSCs. While a ciliated epithelium was observed, there was no GFP expression, indicating that the cells originated from host tissue.

The regeneration and replacement of long segment circumferential defects using pluripotent stem cells has been challenging due to the limited availability of appropriate grafts for tracheal reconstruction, difficulties in whole trachea recellularization procedures, and challenges in deriving epithelial cells in the desired yield and purity. However, the summary of the studies showing the feasibility of using PSCs to generate smaller segments of transplantable tissue is promising [24,77,78,98,168,169]. We define smaller segments as non-circumferential biomaterial-based partial segments, composed of a natural or synthetic scaffold, on which a PSC-derived epithelium is cultured. These studies provide proof-of-concept data for future use in long segment tracheal transplantation applications. In certain cases, for example airway patches generated by our group [166], these smaller constructs have their own clinical utility. They are easier to fabricate and can be specifically used for the repair of damaged tissue requiring the replacement of the epithelium (Figure 4). Moreover, they can be the stepping stone to the generation of larger functional airway sheets which can, for instance, be applied to line the luminal surfaces of bioengineered long-segment circumferential tracheal grafts [21].

## 9. Challenges with iPSCs and Future Directions

While progress has clearly been achieved, the bioengineering of tracheal grafts has several inherent challenges that need continued efforts. These include the reconstitution of a functional cartilage and epithelium, biocompatibility and structural integrity, effective integration, and long-term stability following transplantation. The application of pluripotent stem cells for tracheal regeneration offers the potential of an unlimited source of cells from which a functional airway epithelium can be derived. Pluripotent-derived epithelial cells are advantageous in that they can be used as an autologous source, i.e., derived from a patient’s own somatic cells and hence non-immunogenic. They can also be genetically engineered to ‘escape’ the immune system, as per the recently generated hypo-immune pluripotent cell lines [170,171,172], and thus reduce the need for lifelong immunosuppressive therapies. There are, however, current limitations which hinder their clinical applicability. Current protocols for directed differentiation to epithelial cells are extremely long, labor-intensive and difficult to scale up [162,173,174,175]. It is also difficult to control the purity and yield of specific cell types [176,177,178]. For example, goblet cells are obtained at very low levels [179]. Research focusing on the optimization of directed differentiation protocols to reduce the time required to reach the desired products is necessary. Optimization efforts in other systems have included the use of biophysical cues, enhanced culture and the addition of small molecules to media formulations [180,181,182]. In the context of the airways, modifications involve the utilization of a 3D culture system (airway organoids) to produce induced basal cells (iBCs) from lung progenitors as well as the differentiation of iBCs into a pseudostratified airway epithelium through ALI culture [40,105]. In addition to challenges associated with their differentiation capability, several regulatory and manufacturing barriers must also be addressed. Major regulatory concerns include the risk of tumorigenicity, potential genomic instability during expansion and reprogramming, and the complexity of large-scale production under GMP conditions [183,184]. To reduce the heterogeneity and tumorigenicity in iPSC-derived cells, differentiated cells can be enriched via sorting strategies, and failsafe systems can eliminate aberrant or potentially tumorigenic cells in vivo [170,185,186]. Combined with optimized differentiation protocols and quality control, these approaches may improve the safety and reproducibility of iPSC-derived products and facilitated clinical translation [187]. Nevertheless, these challenges highlight the importance of developing universal pluripotent stem cells, offering off-the-shelf and immune-compatible tracheal graft.

In spite of the successful orthotopic transplantation of long-segment tracheal grafts in rodents [60,188], the surgery presents several challenges including grafts transplantation complexity, especially when involving a fully circumferential trachea. Depending on their anatomical constraints, this animal’s trachea may be too small and complicated to model accurately. On the other hand, anatomical and physiological differences between small animals and humans can affect the translation of the findings to human applications. Our prior research demonstrated that cells seeded on the graft facilitated the regeneration of the epithelium, reducing respiratory complications like stenosis and infection [25]. Nevertheless, the survival of animal models was assessed over a short period (days, weeks). Hence, future investigations should focus on examining the regenerative capabilities of tracheal grafts through long-term monitoring in animal models. Specifically, the assessment should prioritize the longevity and functionality of transplanted epithelial cells, particularly in relation to airway epithelium regeneration.

For the initial feasibility to use iPSCs, prior to processing to a larger scale involving non-economical large animals, a bioreactor offers a controlled and dynamic environment that replicates the physiological conditions necessary for the development and maturation of tissue constructs [18,19,189]. Depending on the specific tissue engineering approach, certain bioreactor designs may be more suitable. For trachea tissue engineering, complex bioreactors equipped with sensors and monitoring tools allow researchers to control various parameters, such as the temperature, pH, and oxygen levels, and assess the tissue development progress in real time. Bioreactors can enhance tracheal graft maturation by providing biochemical cues and mechanical stimulation and optimized nutrient flow for cartilage and epithelial cells proliferation and differentiation and develop biomechanically appropriate trachea constructs. An efficient bioreactor can ensure the uniform seeding and distribution of cells throughout the scaffold, resulting in a more homogeneous and functional tissue construct [19]. Focusing on the development and utilization of complex bioreactor systems, which allow mimicry of the dynamic microenvironments (i.e., breathing, and fluid flow), will be essential for supporting the generation of bioengineered long-segment tracheal constructs. Although this review focuses on epithelial regeneration using stem-cell-derived airway epithelial cells, vascularization remains a critical challenge in tracheal bioengineering. The lack of rapid blood supply in long-segment tracheal grafts can lead to graft ischemia, necrosis, and epithelial loss, which has shown graft failure in previous clinical attempts. Recent strategies such as the pre-vascularization of engineered constructs prior to implantation [190] and the controlled release of angiogenic growth factors (e.g., vascular endothelial growth factor (VEGF) [191], stromal cell-derived factor-1α (SDF-1α), transforming growth factor-β (TGF-β) [192] and endothelial-derived exosomes [193]) have shown the ability to promote rapid angiogenesis. Combining these approaches with epithelial and cartilage regeneration may improve the clinical translation of engineered tracheal grafts. Despite substantial progress, the successful clinical translation of stem cell-based tracheal regeneration will require continued advances in stem cell biology and the generation of a functional epithelium, adequate vascularization, biomaterial design, and long-term graft integration.

## 10. Conclusions

Despite challenges, trachea tissue engineering holds significant promise for revolutionizing the field of respiratory medicine and our ability to construct functional tracheal substitutes benefiting from pluripotent stem cell-derived cells. iPSCs can differentiate into epithelial cells, chondrocytes, and smooth muscle cells, thus allowing the regeneration of a functional epithelium using recipient-derived cells. In addition, recent advancements were made in emerging immune modulation technologies opening possibilities for the use of non-immunogenic cells, creating the potential for universal, off-the-shelf grafts with a reduced risk of immune rejection and improved long-term survival. Together, these technologies are paving the way for the eventual generation of off-the-shelf bioengineered tracheal grafts for effectively treating patients in need of tracheal transplants.

## Data Availability

All data relating to this manuscript is included within the article.
